# Health Effects of Infant Formula Supplemented with Probiotics or Synbiotics in Infants and Toddlers: Systematic Review with Network Meta-Analysis

**DOI:** 10.3390/nu14235175

**Published:** 2022-12-05

**Authors:** Flavia Indrio, Pedro Gutierrez Castrellon, Yvan Vandenplas, Ener Cagri Dinleyici, Ruggiero Francavilla, Massimo Pettoello Mantovani, Assunta Grillo, Isadora Beghetti, Luigi Corvaglia, Arianna Aceti

**Affiliations:** 1 Department of Medical and Surgical Science Pediatric Section, University of Foggia, 71100 Foggia, Italy; 2 Centro de Investigación Translacional en Ciencias de la Salud, Hospital General Dr. Manuel Gea González, 14080 Ciudad de México, Mexico; 3 UZ Brussel, KidZ Health Castle, Vrije Universiteit Brussel, 1090 Brussels, Belgium; 4 Department of Pediatrics, Faculty of Medicine, Eskisehir Osmangazi University, Eskisehir 26480, Turkey; 5 Interdisciplinary Department of Medicine, Pediatric Section, Children’s Hospital ‘Giovanni XXIII’, University of Bari Aldo Moro, 70126 Bari, Italy; 6 Department of Medical and Surgical Sciences, University of Bologna, 40138 Bologna, Italy; 7 Neonatal Intensive Care Unit, IRCCS AOUBO, 40138 Bologna, Italy

**Keywords:** supplemented probiotics, synbiotics, infant formula, infants and toddlers

## Abstract

Supplementation of infant and follow-up formula with probiotics or synbiotics has become a common practice. In 2011 and 2017, the evidence regarding the impact of these interventions was analysed systematically. Recently new evidence was published. To evaluate through a systematic review with network meta-analysis the evidence on the impact of infant formula supplemented with probiotics or synbiotics for healthy infants and 36-month-old toddlers. RCTs published between 1999–2019 for infant formulas supplemented with probiotics alone or synbiotics in healthy infants and toddlers were identified. Data analysis included clinical (gastrointestinal symptoms, risk reduction of infectious diseases, use of antibiotics, weight/height gain and frequency of adverse events) and non-clinical outcomes (changes in faecal microbiota and immune parameters). A random effect model was used. Hedges’ standard mean difference (SMD) and risk ratio (RR) were calculated. Rank analysis was performed to evaluate the superiority of each intervention. Twenty-six randomised controlled trials with 35 direct comparisons involving 1957 children receiving probiotic-supplemented formula and 1898 receiving control formula were reviewed. The mean duration of intervention was 5.6 ± 2.84 months. Certain strains demonstrated a reduction in episodes of colic, number of days with fever and use of antibiotics; however, there was considerable heterogeneity which reduced the level of certainty of effect. No significant effects were observed on weight, height or changes in faecal proportions of *Bifidobacteria, Lactobacillus, Bacteroides* or *Clostridia*. Although there is some evidence that may support a potential benefit of probiotic or synbiotic supplementation of infant formulas, variation in the quality of existing trials and the heterogeneity of the data preclude the establishment of robust recommendations.

## 1. Introduction

The gut microbiota (GM) plays a significant role in several aspects of human health and metabolism. The composition of GM undergoes profound changes during the first 2–3 years of life and again in the elderly. The first contact of newborn babies with microbes is paramount in establishing the type of infant GM which is a key determinant of the overall health status and immunity later in life [1,2]. For a long time, it was believed that the first contact with bacteria happens during the delivery, with children born vaginally having more abundant and diverse gut microbiota, opposite to children delivered by C-section. Some recent studies have challenged this concept by demonstrating the presence of bacteria or bacterial nucleic acids in the umbilical cord blood, amniotic fluid, and placenta with no evidence of chorioamnionitis [3].

Among the contributors to GM assembly, early feeding and the type of feeding is a key factor that modulates the composition and function of gut microbiota tremendously. Mother’s milk contains more than 200 oligosaccharides that are fermented in the colon and stimulate the growth of specific faecal bacteria. In addition to this “prebiotic effect”, the mother’s milk is also a source of live bacteria, including staphylococci, streptococci, bifidobacteria, lactic acid bacteria, beneficial viruses and even fungi (human milk mycobiome) [4,5].

Infants fed with infant formula have, in general, different patterns of gut microbiota in terms of abundance and diversity, with evidence showing that infants and toddlers fed under this strategy had different responses to infection episodes (diarrhoea and respiratory infections). The microbiota of formula-fed infants is more similar to that of adults, consisting of genes related to bile acid synthesis and methanogenesis [6].

These differences in microbiota between human milk-fed and formula-fed babies motivated researchers to find strategies aimed at modulating the microbiota pattern to obtain in formula-fed babies similar patterns to human milk-fed babies. Supplementation of infant and follow-on formulas (IF) with probiotics (single or multi-strain) and prebiotics has become common practice in the food industry. In 2011 the Committee of Nutrition of the European Society of Gastroenterology, Hepatology and Nutrition (ESPGHAN) published a systematic review of the evidence on the safety and efficacy of the use of formulas supplemented with probiotics. Although no negative impacts on infant growth or other related adverse events were observed, there was insufficient evidence to recommend routine use [7]. In 2017, an updated systematic review was published, identifying adjusted effects according to the strain or combination of strains for reduction of the number of gastrointestinal or respiratory infections, reduction in the number of episodes of colic or regurgitation, a higher frequency of bowel movements and in some cases accelerated growth [8]. However, the absence of high-quality evidence precluded a strong recommendation from being made. Considering new evidence becoming available [9,10,11,12,13,14,15,16,17,18,19,20,21,22,23,24,25], the aim of this review was to evaluate the effect on the clinical and non-clinical outcome of infant formula supplemented with probiotics or synbiotics, as compared to placebo or different probiotic strains, for healthy infants and 36-month-old toddlers using network meta-analysis (NMA) approach.

## 2. Materials and Methods

### 2.1. Study Protocol and Search Strategy

This systematic review was conducted following The PRISMA Extension Statement for Reporting of Systematic Reviews Incorporating Network Meta-analyses of Health Care Interventions [26,27]. We only included double-blind RCT published between January 1999 and December 2019 and written in the English language. A systematic and exhaustive search was conducted in Medline, Embase, Cumulative Index to Nursing and Allied Health (CINAHL), PsycINFO, the Cochrane Central Register of Controlled Trials, Lilacs, Artemisa and in the databases of the principal international regulatory agencies. A systematic and sensitive validated strategy was used to identify the best evidence (Appendix A).

### 2.2. Study Selection and Outcome Measures

RCTs that compared the use of infant formula and follow-on formula with probiotics alone or with synbiotics added during the manufacturing process vs infant and follow-on formula without probiotics and/or human milk in healthy infants or toddlers (36 months) were selected for this NMA. RCT reporting supplementation with both probiotics and prebiotics was kept in the analysis. All interventions were included in nodes and compared to placebo as the standard of reference. Data analysis included clinical (gastrointestinal symptoms, risk reduction of infectious diseases, use of antibiotics, weight/height gain and frequency of adverse events) and non-clinical outcomes (changes in faecal microbiota and immune parameters).

### 2.3. Data Extraction and Quality Analysis

The risk of bias was evaluated according to the Cochrane approach [28]. Any discrepancy in the evaluation of the articles was resolved using the Delphi methodology, which was coordinated by PGC. Analysed data included age, sample size, type and dose of probiotic, duration of intervention, use of prebiotics and reported outcomes.

### 2.4. Data Synthesis and Analysis

The statistical strategy used a multiple-treatment meta-analysis. Considering that the majority of studies compared the use of infant formula with no probiotics (control infant formula), we decided to use this intervention as the central axis for direct comparisons. Dichotomous outcomes were analysed with the total number of randomly assigned participants as the denominator. For the secondary analysis of efficacy, measured as a binary outcome, the outcomes for missing information were generated, assuming that all participants with missing data did not respond to treatment. When reported, information on participants that discontinued the intervention was included in the analysis. For each potentially eligible study, descriptive statistics of the population characteristics and their results were reported, describing the type of comparison as well as the most important clinical and methodological variables. For each pairwise comparison (direct or indirect), Hedges’ standard mean difference (SMD) was calculated for continuous numeric variables, whereas the respective risk ratio (RR) was calculated for dichotomous outcomes. Both were calculated with their respective 95% confidence interval (CI95%). The first meta-analysis was a paired comparison of all published studies. We used a random effect model, considering that different studies estimated different treatment effects. Concomitantly, we calculated I^2^ for heterogeneity and its corresponding *p*-value. Thereafter we assembled an NMA using a random effect model with a Bayesian approach [29,30] and summarised the results using effect sizes and CI95%. We used the adjusted model as described by Salanti et al. [31]. Additionally, we calculated the probability of superiority for each “anti-colic” intervention through a SUCRA analysis and presented the results in a ranked graph as described by Salanti et al. [32]. To estimate the inconsistency (discordance between direct and indirect evidence with a CI95% that did not include zero), we calculated the difference between the direct and indirect estimates, taking as reference only the constructed indicators that had included a placebo group [33]. Finally, we adjusted the model with and without assumptions of consistency and compared the two models in terms of fit and parsimony [34]. In the case of a significant inconsistency, we investigated the distribution of clinical and methodological variables that might have been a potential source of heterogeneity or inconsistency in each group of specific comparisons. All analysis and graphic depictions were performed on version 16 of STATA for Mac.

## 3. Results

**Figure 1:**
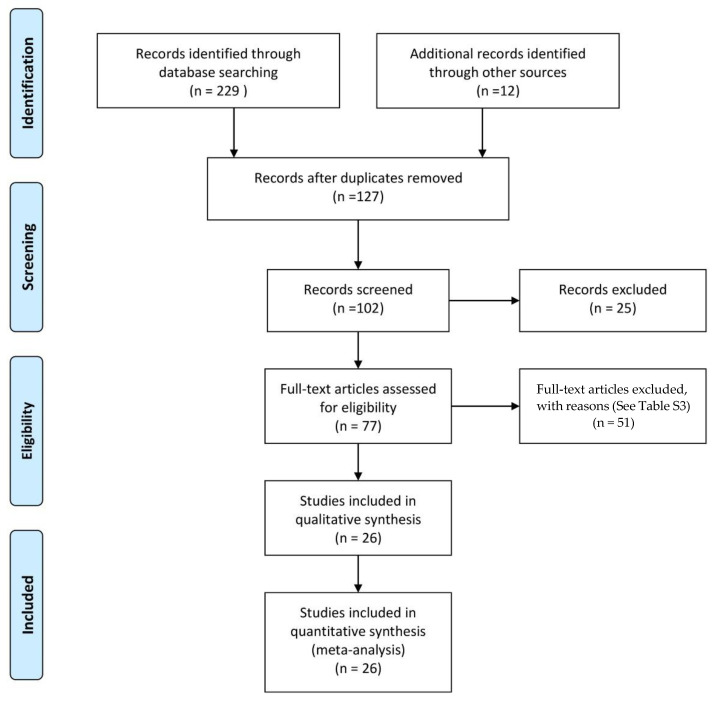
Flow diagram of the search strategy and study selection.

After quality evaluation, 26 RCTs [9,12,13,18,21,22,23,35,36,37,38,39,40,41,42,43,44,45,46,47,48,49,50,51,52,53] were considered for full analysis (Figure 1).

Twenty-nine different probiotic comparisons were analysed; 21 were combinations of *Bifidobacteria* and 8 of *Lactobacilli* (Supplemental Table S1). The evaluation of the quality of the evidence and the reasons for excluding the articles are included in Supplemental Tables S2 and S3. A total of 1898 and 1957 children, one day to 36 months old, were assigned to placebo vs probiotic or probiotics/prebiotic infant formula, respectively. The mean duration of the interventions was 5.6 ± 2.84 months (3 to 12 months). Details of the included studies are reported in Table 1.

### 3.1. Clinical Outcomes

#### 3.1.1. Functional Gastrointestinal Disorders (FGDs)

**Figure 2:**
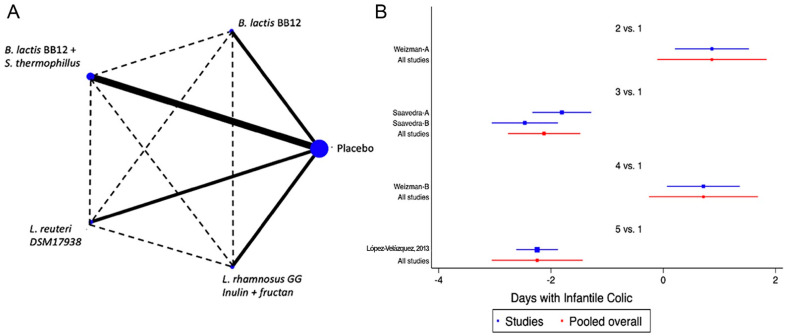
Network Meta-Analysis for probiotics in infant formula and colic. (**A**) Network diagram: the area of the blue nodes is based on the total number of patients for each respective interventions, the thickness of continuous lines represents the total number of studies comparing treatments/nodes vs placebo, dashed lines represent comparisons between two active interventions; (**B**) forest plot of multiple treatments.

Eight of the studies included in the analysis evaluated at least one of the FGDs observed in infants (colic, regurgitation or functional constipation) [18,37,38,40,44,47,49,50]. For infantile colic, the clinical parameter of crying time per day was considered. The analysed probiotics were *Bifidobacterium lactis* BB12 [34], a combination of *B. lactis* BB12 with *Streptococcus thermophilus* [40], *Lactobacillus reuteri* (*L. reuteri*) DSM 17938 [38] and *L. rhamnosus* GG (LGG) combined with inulin and fructans [49]. When probiotics were analysed as a group, a positive impact was identified [SMD of −1.42 days, Confidence Interval 95% (CI95%) −1.66 to −1.19, *p* < 0.05, I2 of 96.7%]. The analysis of the information through the NMA, confirmed the positive effect was predominantly for LGG combined with inulin and fructans or the combinations of *B. lactis* BB12 with *Streptococcus thermophilus* (Figure 2 and Figure S1). For regurgitation, three trials were identified, one with *L. reuteri* [50], one with LGG combined with inulin and fructans [49], and one with *B. lactis* strain CNCM-I −3446 combined with BMOS [18], observing a global positive impact on the reduction of the number of regurgitation episodes (SMD −3.47, CI95% −3.73 to −3.21, *p* < 0.05, I^2^ 96.3%), with very similar effects between different strains of probiotics. In the case of functional constipation, only a single clinical trial was identified, in which *L. salivarius* CECT5713 2 × 10^6^ CFU/g was used in 40 children vs placebo, without identifying significant differences between the groups [47].

#### 3.1.2. Reduction of the Risk for Infections, Use of Antibiotics, Days with Fever

**Figure 3:**
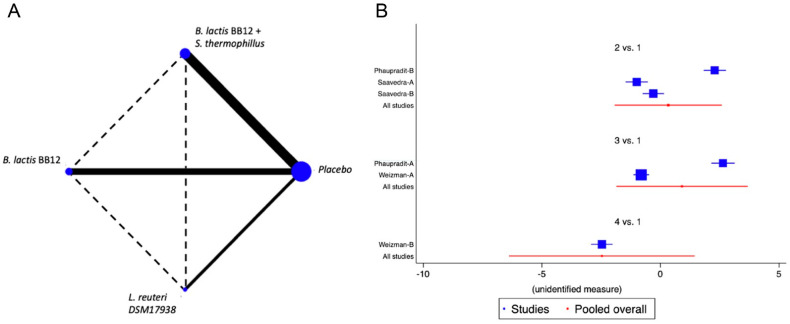
Network Meta-Analysis for probiotics in infant formula and diarrhoea. (**A**) Network diagram: the area of the blue nodes is based on the total number of patients for each respective interventions, the thickness of continuous lines represents the total number of studies comparing treatments/nodes vs placebo, dashed lines represent comparisons between two active interventions; (**B**) forest plot of multiple treatments.

Five trials were identified [18,21,35,38,47] with seven different combinations, in which the reduction in the number of diarrhoea episodes was evaluated. The probiotics used were *B. longum BL999, B. lactis, B. infantis, B. breve and L. fermentum*, identifying a marginal effect on the reduction in the number of episodes, with an overall relative risk (RR) of 0.85, CI95% of 0.75 to 1.02, *p* NS, I2 75%. The NMA did not identify the superiority of any of the analysed strains. Reduction in the duration of diarrheal events was analysed in four studies [18,35,38,47] with six different combinations of *B. lactis BB12, B. lactis BB12 with S. thermophilus, or L. reuteri* DSM 17938. No positive impact on this outcome was identified in the global analysis nor in the NMA for any specific strain (Figure 3 and Figure S2).

**Figure 4:**
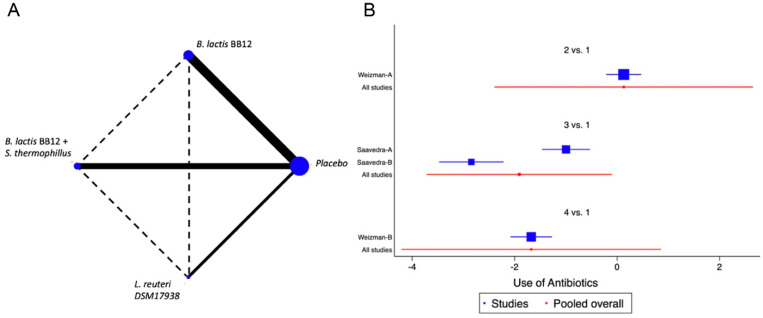
Network Meta-Analysis for probiotics in infant formula and use of antibiotics. (**A**) Network diagram: the area of the blue nodes is based on the total number of patients for each respective interventions, the thickness of continuous lines represents the total number of studies comparing treatments/nodes vs placebo, dashed lines represent comparisons between two active interventions; (**B**) forest plot of multiple treatments.

When we evaluated the impact of the interventions on days with respiratory tract infections, a single study was identified [38]. *B. lactis* BB12 was compared with *L. reuteri* DSM17938 or placebo. A marginal effect in reducing episodes was observed (SMD −0.27, CI95% −0.52 to −0.02, *p* 0.03, I^2^ 93%) in favour of *L. reuteri,* although the heterogeneity was so significant to establish recommendations. The effect on fever was evaluated on the same RCT that analysed respiratory infections [38]. A positive impact in the reduction of days with fever was observed, although there was significant heterogeneity (SMD −0.83, CI95% −1.10 to −0.56, *p* < 0.05, I^2^ 98%). On the reduction of the use of antibiotics, two trials were identified [37,38], with four combinations of probiotics, *B. lactis* BB12 or *L. reuteri* [37] and *B. lactis* BB12 combined with *S. thermophilus* [37], identifying an overall positive impact in the reduction of the use of antibiotics (SMD −0.96, CI95% −1.17 to −0.75, *p* < 0.05, I^2^ 96.5%). The NMA allowed us to identify that the effect was predominantly due to the combination of *B. lactis* BB12 with *S. thermophilus*, although with a significant level of bias (Figure 4 and Figure S3).

#### 3.1.3. Growth Parameters

**Figure 5:**
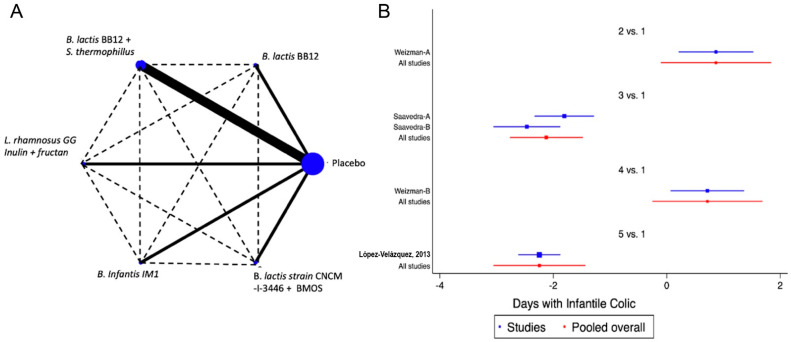
Network Meta-Analysis for probiotics in infant formula and change on weight/height Z score. (**A**) Network diagram: the area of the blue nodes is based on the total number of patients for each respective interventions, the thickness of continuous lines represents the total number of studies comparing treatments/nodes vs placebo, dashed lines represent comparisons between two active interventions; (**B**) forest plot of multiple treatments.

A total of 10 trials [18,36,37,42,44,46,47,48,49,52] were identified that assessed the impact of the interventions on weight gain, height gain, and change in W/H Z Score values as outcomes. No significant impact of the interventions on these outcomes was identified (SMD −0.02, CI95% −0.15 to 0.10, *p* NS; I^2^ 93%). Similarly, the NMA did not allow the identification of the impact of the different combinations of probiotics on these parameters (Figure 5 and Figure S4).

### 3.2. Non-Clinical Outcomes

#### 3.2.1. Immunological Parameters

Two studies [22,35] using *B. lactis* BB12, *B. lactis* BB12 combined with *S. thermophilus*, or the combination of *B. infantis* R0033, *B. bifidum* R0071 and *L. helveticus* R0052, evaluated the changes on the levels of salivary IgA (SIgA). Unfortunately, in two of the studies, only the mean value was reported, so it was not possible to incorporate them in the meta-analysis (Supplemental Table S3).

#### 3.2.2. Change in Faecal Microbiota

**Figure 6:**
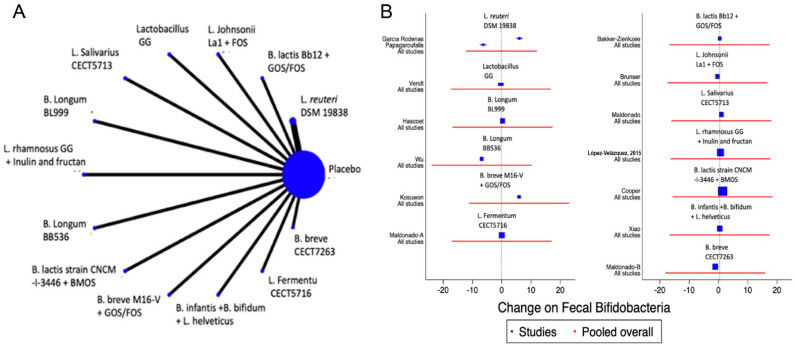
Network meta-analysis for probiotics and change on faecal *Bifidobacteria*. (**A**) Network diagram: the area of the blue nodes is based on the total number of patients for each respective interventions; (**B**) forest plot of multiple treatments.

**Figure 7:**
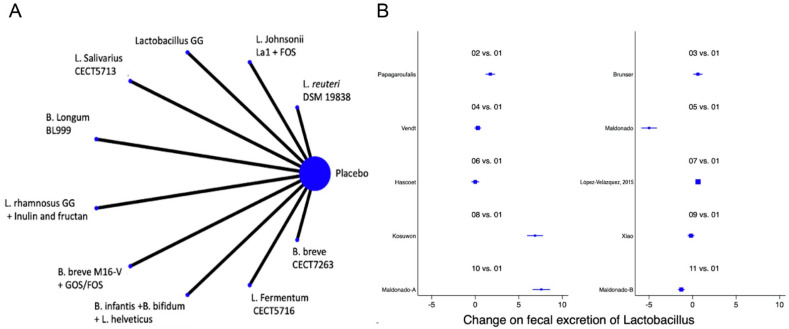
Network meta-analysis for probiotics and change on faecal *Lactobacillus*. (**A**) Network diagram: the area of the blue nodes is based on the total number of patients for each respective interventions; (**B**) forest plot of multiple treatments.

A total of 13 studies were identified [12,13,22,23,39,41,42,47,48,50,51,52,53] with a total of 14 comparisons evaluating the impact of the interventions on the change in the faecal abundance of *Bifidobacteria, Lactobacilli, Enterobacteriaceae, Bacteroides* or Clostridium, with a total of 14 comparisons. Overall, no significant changes in the composition of faecal microbiota in terms of abundance or diversity were identified (Figure 6 and Figure 7 and Supplemental Figures S5 and S6).

## 4. Discussion

This NMA demonstrate, based on the current literature, some isolated effects of the use of probiotics added to infant formula, mainly in terms of a modest reduction in the frequency or severity of colic or regurgitation. Some strains demonstrated a reduction in episodes of colic, number of days with fever and use of antibiotics; however, there was considerable heterogeneity which reduced the level of certainty of effect. Although the total number of 26 RCTs included with over 1957 infants is considerable, many of the different probiotic treatments were only evaluated in 1 or 2 trials.

Regarding the effect of IF added with probiotics in FGDS, the majority of the studies evaluated the effect on infantile colic. The evaluated probiotics were *Bifidobacterium lactis BB12* (1 study), a combination of *B. lactis BB12* with *Streptococcus thermophilus* (2 studies), *Lactobacillus* (now *Limosilactobacillus*) *reuteri* DSM 17938 (1 study) and Lactobacillus (now Lacticaseibacillus) rhamnosus GG (LGG) combined with inulin and fructan-derived from agave (1 study). In functional regurgitation, three trials were identified, using L. reuteri (one study), *Lactobacillus GG* combined with inulin and fructan agave-derived (one study), and *B. lactis strain CNCM-I −3446* combined with BMOS.

In the case of functional constipation, only a single clinical trial was identified, in which *L. salivarius CECT5713* 2 × 10^6^ CFU/g was used in 40 children vs placebo, without identifying significant differences between the groups. Four trials were identified, with 7 different combinations, in the evaluation of the reduction in the number of diarrhoea episodes. The probiotics used were *B. longum BL999, B. lactis, B. infantis, B. breve* and *L. fermentum*, identifying a marginal effect on the reduction in the number of episodes. The NMA did not identify the superiority of any of the analysed strains. When we evaluated the impact of the interventions on days with respiratory tract infections, a single study was identified in which the use of *B. lactis BB12* was compared in one group of infants vs placebo and in another intervention branch L. reuteri DSM17938. A considerable effect in reducing episodes was observed (SMD −0.27, CI95% −0.52 to −0.02, *p* 0.03, I^2^ 93%), with a greater effect in favour of *L. reuteri*. The same effect was observed for days with fever. This outcome was evaluated in the same studies reported for respiratory infections, identifying a positive impact in the reduction of days with fever, although there was significant heterogeneity (SMD −0.83, CI95% −1.10 to −0.56, *p* < 0.05, I^2^ 98%). The positive effect observed was in favour of *L. reuteri*. On the use of antibiotics, three trials were identified with four combinations of probiotics, *B. lactis BB12* (one study), *B. lactis BB12* combined with *S. thermophilus* (two studies) and L. reuteri (one study), identifying an overall positive impact in the reduction of the use of antibiotics (SMD −0.96, CI95% −1.17 to −0.75, *p* < 0.05, I^2^ 96.5%). The NMA allowed us to identify that the effect was predominantly due to the combination of *B. lactis BB12* with *S. thermophilus*, although with a significant level of bias. Regarding immunity, three studies were identified that evaluated changes in the levels of salivary IgA (SIgA) using B. lactis BB12, B. *lactis BB12* combined with *S. thermophilus,* or the combination of *B. infantis R0033, B. bifidum R0071 and L. helveticus R0052*. Unfortunately, in two of the studies, the mean value was reported without the SDs, so it was not possible to incorporate them in the meta-analysis.

For the analysis of the impact of probiotics in infant formula and the effect on growth parameters, a single trial of *L. reuteri* DSM 17938 showed a positive effect.

Finally, regarding changes in faecal microbiota, a total of 10 studies analysed potential changes in the faecal populations of *Bifidobacteria, Lactobacilli, Enterobacteriaceae, Bacteroides or Clostridium*. Overall, no significant change was identified in abundance or diversity. Some limitations of this NMA include the lack of an adequate number of RCTs on the various analysed outcomes to be on the possibility to establish strong recommendations, the different scales or evaluation tools used to measure the different outcomes, which make it hard to pool different results on weighted analysis and the number of children included in some trials which reduced the power and probability to identify reals effects. A further possible limitation needs to be acknowledged: actually, both breastfed and formula-fed infants were included in this analysis in the control group. However, the clinical question of the study was to assess clinical and non-clinical outcomes of infant formula supplemented with probiotics compared to “standard” feeding options for healthy term infants and toddlers (i.e., standard formula or mothers’ own milk/breastfeeding).

To conclude, our efforts in this study were to present an overview of all published evidence on the use of probiotics in infant formula.

We believe that our approach of a strain-specific NMA gives a much more meaningful answer than previously performed meta-analyses.

Our study, which updated previous systematic reviews, shows that even though we identified some isolated effects of the use of probiotics added to infant formula, mainly in terms of a modest reduction in the frequency or severity of colic or regurgitation, the number of studies and related heterogeneity does not allow to make definitive recommendations. The same occurred with the potential effects on other clinical outcomes such as infection protection (gastrointestinal or respiratory infections) and reduction in the use of antibiotics

Although there is some evidence that may support a potential benefit of probiotic supplementation of infant formulas, variation in the quality of existing trials and the heterogeneity of the data preclude the establishment of robust recommendations

There is a need to analyse potential modes of action and how specific strains are playing a significant role in the observed limited clinical effects.

## Figures and Tables

**Table 1:** The main characteristics of the randomised controlled trials were included in the network meta-analysis.

Author	Year	Ages	n1	n2	n. HumanMilk	Intervention	Follow-Up (Months)
Phuapradit-A [35]	1999	6 to 36 m	62	57	NA	*B. lactis* Bb12 (10^8^ cfu/g)	8
Nopchinda-A [36]	2002	6 to 36 m	51	43		*B. lactis* Bb12 (3 × 10^7^ cfu/g)	6
Weizman-A [38]	2005	4 to 10 m	73	60	NA	*B. lactis* Bb12 1 × 10^7^ cfu/g	3
Weizman-A [40]	2006	4 m	20	19	NA	*B. lactis* Bb12 1 × 10^7^ cfu/g	3
Phuapradit-B [35]	1999	6 to 36 m	56	57	NA	*B. lactis* Bb12 + *S. thermophilus* (dose not reported)	8
Nopchinda-B [36]	2002	6 to 36 m	54	43	NA	*B. lactis* Bb12 + *S. thermophilus* (3 × 10^7^ cfu/g)	6
Saavedra-A [37]	2004	3 to 24 m	39	40	NA	*B. lactis* Bb12 + *S. thermophilus* 1 × 10^7^ cfu/g	6
Saavedra-B [37]	2004	3 to 24 m	39	40	NA	*B. lactis* Bb12 + *S. thermophilus* 1 × 10^6^ cfu/g	6
Weizman-B [38]	2005	4 to 10 m	68	60	NA	*L. reuteri* 1 × 10^7^ cfu/g	3
Weizman-B [40]	2006	4 m	20	19	NA	*L. reuteri* 1 × 10^7^ cfu/g	3
Papagaroufalis [50]	2014	3 days	36	35	NA	*L. reuteri* 1.2 × 10^9^ cfu/L	3
Garcia Rodenas [12]	2016	3 days	36	35	NA	*L. reuteri* DSM 17938 1.2 × 10^6^ cfu/mL	5
Bakker-Zierikzee [39]	2005	3 days	19	19	63	*B. lactis* Bb12 6 × 10^9^ cfu/100 mL + GOS/FOS (6 g/L; 90%/10%)	4
Bakker-Zierikzee [39]	2005	3 days	19	19	63	*B. lactis* Bb12 6 × 10^9^ cfu/100 mL + GOS/FOS (6 g/L; 90%/10%)	8
Brunser [41]	2006	3 to 5 months	25	33	26	*L. johnsonii La1* 10^8^ cfu/g + FOS	3
Vendt [42]	2006	2 months	51	54	NA	*Lactobacillus* GG 10^7^ cfu/g	6
Mah [43]	2007	<14 dias	20	17	NA	*B. longum* BB536 1 × 10^7^ cfu/g + LGG 2 × 10^7^ cfu	12
Chouraqui-A [44]	2008	<14 dias	29	25	NA	*B. longum* BL999 (1.29 × 10^8^ cfu/100 mL) + *Lactobacillus paracasei* ST11 (6.45 × 10^8^ cfu/100 mL)	*4*
Chouraqui-B [44]	2008	<14 dias	28	25	NA	*B. longum* BL 999 1.29 × 10^8^ cfu/100 mL) + *L. paracasei* ST11 (6.45 × 10^8^ cfu/100 mL) + GOS/FOS (0.4 g/100 mL; 90%/10%)	4
Chouraqui-C [44]	2008	<14 dias	28	25	NA	*B. longum* BL (2.58 × 10^8^ cfu/100 mL) + *L. paracasei* ST11 (2.58 × 10^8^ cfu/100 mL + GOS/FOS (0.4 g/100 mL; 90%/10%)	4
Haschke-Becher [45]	2008	4 meses	17	18	23	*L. johnsonii La1* 10^8^ cfu/g	6
Gibson [46]	2009	<10 days	72	70	NA	*B. lactis* Bb12 3.85 × 10^8^ cfu + DHA and AA	7
Maldonado [47]	2010	6 months	40	40	NA	*L. salivarius* CECT5713 2 × 10^6^ cfu/g	6
Hascoet [48]	2011	<7 days	40	38	73	*B. longum* BL999 2 × 10^7^ cfu/g	4
López-Velázquez [49]	2013	<14 days	93	89	147	*L. rhamnosus* GG 1 × 10^7^ CFU/g + Inulin and fructan from agave (0.5g/100mL)	4
López-Velázquez [51]	2015	<14 days	93	89	147	*L. rhamnosus* GG 1 × 10^7^ CFU/g + Inulin and fructan from agave (0.5 g/100 mL)	3
Wu [13]	2016	0–7 days	129	135	NA	*B. longum* BB536 1 × 10^7^ cfu/g	12
Cooper [52]	2016	0–3 days	217	213	NA	*B. lactis* strain CNCM-I-3446, 1 × 10^7^ cfu/g + BMOS (GOS + 3′- and 6′-sialyllactose, 8 g/L)	6
Radke [18]	2017	<14 days	169	160	51	*B. lactis* strain CNCM-I-3446, 1 × 10^7^ cfu/g + BMOS (GOS + 3′- and 6′-sialyllactose, 8 g/L)	6
Escribano [21]	2018	<3 months	73	78	NA	*B. infantis* IM1 (107 cfu/g)	3
Kosuwon [53]	2018	1–3 years	65	64	NA	*B. breve* M16-V (1.8 × 10^7^ CFU/g) + GOS/FOS (9.5 g/L; 90%/10%)	3
Xiao [22]	2019	3 to 6 months	48	57	NA	1.425 × 10^8^ cfu of each *B. infantis* R0033 and *B. bifidum* R0071, with 9.6 × 10^9^ cfu of *L. helveticus* R0052	3
Maldonado-A [23]	2019	<30 days	65	61	NA	*L. fermentum* CECT5716 Lc40 (10^7^ cfu/g)	12
Maldonado-B [23]	2019	<30 days	63	61	NA	*B. breve* CECT7263 (10^7^ cfu/g)	12

## Data Availability

Not applicable.

## Supplementary Materials

The following supporting information can be downloaded at: https://www.mdpi.com/article/10.3390/nu14235175/s1, Table S1: Type of Probiotics included in the analysis; Table S2: Risk of bias evaluation for included RCTs; Table S3: Excluded randomised controlled trials; Supplemental Figure S1: Network Meta-Analysis for probiotics in infant formula and colic, comparison-adjusted funnel plot of multiple treatments; Supplemental Figure S2: Network Meta-Analysis for probiotics in infant formula and diarrhoea, comparison-adjusted funnel plot of multiple treatments; Supplemental Figure S3: Network Meta-Analysis for probiotics in infant formula and use of antibiotics, comparison-adjusted funnel plot of multiple treatments; Supplemental Figure S4: Network Meta-Analysis for probiotics in infant formula and change on weight/height Z score, comparison-adjusted funnel plot of multiple treatments; Supplemental Figure S5: Network meta-analysis for probiotics and change on faecal *Bifidobacteria*, comparison-adjusted funnel plot of multiple treatments; Supplemental Figure S6: Network meta-analysis for probiotics and change on faecal *Lactobacilli*, comparison-adjusted funnel plot of multiple treatments.

## Appendix A. Systematic and Sensitive Validated Strategy for Searching Evidence

Pubmed searching algorithms included the terms ‘ randomised controlled trial’ OR random*:de,ab,ti OR factorial*:de,ab,ti OR crossover*:de,ab,ti OR (cross NEXT/1 over*):de,ab,ti OR placebo*:de,ab,ti OR (doubl* NEAR/1 blind*):de,ab,ti OR (singl* NEAR/1 blind*):de,ab,ti OR assign*:de,ab,ti OR allocat*:de,ab,ti OR volunteer*:de,ab,ti AND (formula OR infant AND formula OR ‘follow on’ AND formula OR supplemented AND formula AND bifidobacterium AND animalis OR streptococcus AND thermophilus OR lactobacillus AND helveticus OR lactobacillus AND johnsonii OR Bifidobacterium AND lactis OR bifidobacterium AND aimalis OR lactobacillus AND rhamnosus OR bifidobacterium AND longum OR lactobacillus AND reuteri OR lactobacillus AND salivarius OR galactooligosaccharides OR scgos OR gos OR fructooligosaccharides OR lcfos OR fos OR acidic AND oligosaccharides OR inulin OR oligofructose OR polydextrose OR probiotic* OR prebiotic* OR ‘pre biotic*’ OR synbiotic*) AND (infant OR infancy OR infants OR newborn OR child OR children NOT sick children).

## References

[1] Li M., Wang M., Donovan S.M. (2014). Early development of the gut microbiome and immune-mediated childhood disorders. Semin. Reprod. Med..

[2] Wang M., Monaco M.H., Donovan S.M. (2016). Impact of early gut microbiota on immune and metabolic development and function. Semin. Fetal. Neonatal. Med..

[3] Derrien M., Alvarez A.-S., deVos W.M. (2019). The gut microbiota in the first decade of life. Trends Microbiol..

[4] Ho N.T., Li F., Lee-Sarwar K.A., Tun H.M., Brown B.P., Pannaraj P.S., Bender J.M., Azad M.B., Thompson A.L., Weiss S.T. (2018). Meta-analysis of effects of exclusive breastfeeding on infant gut microbiota across populations. Nat. Commun..

[5] Savage J.H., Lee-Sarwar K.A., Sordillo J.E., Lange N.E., Zhou Y., O’Connor G.T., Sandel M., Bacharier L.B., Zeiger R., Sodergren E. (2018). Diet during pregnancy and infancy and the infant intestinal microbiome. J. Pediatr..

[6] Forbes J.D., Azad M.B., Vehling L., Tun H.M., Konya T.B., Guttman D.S., Field C.J., Lefebvre D., Sears M.R., Becker A.B. (2018). Association of exposure to formula in the hospital and subsequent infant feeding practices with gut microbiota and risk of overweight in the first year of life. JAMA Pediatr..

[7] Braegger C., Chmielewska A., Decsi T., Kolacek S., Mihatsch W., Moreno L., Piescik M., Puntis J., Shamir R., Szajewska H. (2011). Supplementation of infant formula with probiotics and/or prebiotics: A systematic review and comment by the ESPGHAN committee on nutrition. J. Pediatr. Gastroenterol. Nutr..

[8] Skórka A., Pieścik-Lech M., Kołodziej M., Szajewska H. (2017). To add or not to add probiotics to infant formulae? An updated systematic review. Benef. Microbes.

[9] Baglatzi L., Gavrili S., Stamouli K., Zachaki S., Favre L., Pecquet S., Benyacoub J., Costalos C. (2016). Effect of Infant Formula Containing a Low Dose of the Probiotic Bifidobacterium lactis CNCM I-3446 on Immune and Gut Functions in C-Section Delivered Babies: A Pilot Study. Clin. Med. Insights Pediatr..

[10] Berni Canani R., Sangwan N., Stefka A.T., Nocerino R., Paparo L., Aitoro R., Calignano A., Khan A.A., Gilbert J.A., Nagler C.R. (2016). Lactobacillus rhamnosus GG-supplemented formula expands butyrate-producing bacterial strains in food allergic infants. ISME J..

[11] Xu L., Wang Y., Wang Y., Fu J., Sun M., Mao Z., Vandenplas Y. (2016). A double-blinded randomized trial on growth and feeding tolerance with Saccharomyces boulardii CNCM I-745 in formula-fed preterm infants. J. Pediatr. (Rio. J.).

[12] Rodenas C.L.G., Lepage M., Ngom-Bru C., Fotiou A., Papagaroufalis K., Berger B. (2016). Effect of Formula Containing Lactobacillus reuteri DSM 17938 on Fecal Microbiota of Infants Born by Cesarean-Section. JPGN.

[13] Wu B.B., Yang Y., Xu X., Wang W.P. (2016). Effects of Bifi dobacterium supplementation on intestinal microbiota composition and the immune response in healthy infants. World, J. Pediatr..

[14] Bazanella M., Maier T.V., Clavel T., Lagkouvardos I., Lucio M., Maldonado-Gòmez M.X., Autran C., Walter J., Bode L., Schmitt-Kopplin P. (2017). Randomized controlled trial on the impact of early-life intervention with bifidobacteria on the healthy infant fecal microbiota and metabolome. Am. J. Clin. Nutr..

[15] Scalabrin D.M.F., Harris C., Johnston W.H., Berseth C.L. (2017). Long-term safety assessment in children who received hydrolyzed protein formulas with Lactobacillus rhamnosus GG: A 5-year follow-up. Eur. J. Pediatr..

[16] Canani R.B., Di Costanzo M., Bedogni G., Amoroso A., Cosenza L., Di Scala C., Granata V., Nocerino R. (2017). Extensively hydrolyzed casein formula containing Lactobacillus rhamnosus GG reduces the occurrence of other allergic manifestations in children with cow’s milk allergy: 3-year mrandomized controlled trial. J. Allerg. Clin. Immunol..

[17] Indrio F., Riezzo G., Giordano P., Ficarella M., Miolla M.P., Martini S., Corvaglia L., Francavilla R. (2017). Effect of a Partially Hydrolysed Whey Infant Formula Supplemented with Starch and Lactobacillus reuteri DSM 17938 on Regurgitation and Gastric Motility. Nutrients.

[18] Radke M., Picaud J.C., Loui A., Cambonie G., Faas D., Lafeber H.N., de Groot N., Pecquet S.S., Steenhout P.G., Hascoet J.M. (2017). Starter formula enriched in prebiotics and probiotics ensures normal growth of infants and promotes gut health: A randomized clinical trial. Pediatr. Res..

[19] Vandenplas Y., Analitis A., Tziouvara C., Kountzoglou A., Drakou A., Tsouvalas M., Mavroudi A., Xinias I. (2017). Safety of a New Synbiotic Starter Formula. Pediatr. Gastroenterol. Hepatol. Nutr..

[20] Candy D.C., Van Ampting M.T., Oude Nijhuis M.M., Wopereis H., Butt A.M., Peroni D.G., Vandenplas Y., Fox A.T., Shah N., West C.E. (2018). A symbiotic containing amino-acid-based formula improves gut microbiota in non-IgE-mediated allergic infants. Pediatr. Res..

[21] Escribano J., Ferré N., Gispert-Llaurado M., Luque V., Rubio-Torrents C., Zaragoza-Jordana M., Polanco I., Codoner F.M., Chenoll E., Morera M. (2018). Bifidobacterium longum subsp infantis CECT7210-supplemented formula reduces diarrhea in healthy infants: A randomized controlled trial. Pediatr. Res..

[22] Xiao L., Gong C., Ding Y., Ding G., Xu X., Deng C., Ze X., Malard P., Ben X. (2019). Probiotics maintain intestinal secretory immunoglobulin A levels in healthy formula fed infants: A randomised, double-blind, placebo-controlled study. Benef. Microbes.

[23] Maldonado J., Gil-Campos M., Maldonado-Lobón J.A., Benavides M.R., Flores-Rojas K., Jaldo R., Jiménez Del Barco I., Bolívar V., Valero A.D., Prados E. (2019). Evaluation of the safety, tolerance and efficacy of 1-year consumption of infant formula supplemented with Lactobacillus fermentum CECT5716 Lc40 or Bifidobacterium breve CECT7263: A randomized controlled trial. BMC Pediatr..

[24] Chi C., Xue Y., Liu R., Wang Y., Lv N., Zeng H., Buys N., Zhu B., Sun J., Yin C. (2020). Effects of a formula with a probiotic Bifidobacterium lactis Supplement on the gut microbiota of low birth weight infants. Eur. J. Nutr..

[25] Li X., Peng Y., Li Z., Christensen B., Heckmann A.B., Stenlund H., Lönnerdal B., Hernell O. (2019). Feeding Infants Formula with Probiotics or Milk Fat Globule Membrane: A Double-Blind, Randomized Controlled Trial. Front. Pediatr..

[26] Moher D., Liberati A., Tetzlaff J., Altman D.G., PRISMA Group (2009). Preferred reporting items for systematic reviews and meta-analyses: The PRISMA statement. BMJ.

[27] Hutton B., Salanti G., Caldwell D.M., Chaimani A., Schmid C.H., Cameron C., Ioannidis J.P., Straus S., Thorlund K., Jansen J.P. (2015). The PRISMA extension statement for reporting of systematic reviews incorporating network meta-analyses of health care interventions: Checklist and explanations. Ann. Intern. Med..

[28] Higgins J.P.T., Thomas J., Chandler J., Cumpstonm M., Li T., Page M.J., Welch V.A. (2019). Cochrane Handbook for Systematic. Reviews of Interventions.

[29] Lu G., Ades A.E. (2004). Combination of direct and indirect evidence in mixed treatment comparisons. Stat. Med..

[30] Ades A.E., Sculpher M., Sutton A., Abrams K., Cooper N., Welton N., Lu G. (2006). Bayesian methods for evidence synthesis in cost-effectiveness analysis. Pharmacoeconomics.

[31] Salanti G., Higgins J.P., Ades A.E., Ioannidis J.P. (2008). Evaluation of networks of randomized trials. Stat. Methods Med. Res..

[32] Salanti G., Ades A.E., Ioannidis J.P. (2011). Graphical methods and numerical summaries for presenting results from multiple-treatment meta-analysis: An overview and tutorial. J. Clin. Epidemiol..

[33] Salanti G., Marinho V., Higgins J.P. (2009). A case study of multiple-treatments meta-analysis demonstrates that covariates should be considered. J. Clin. Epidemiol..

[34] Dias S., Welton N.J., Caldwell D.M., Ades A.E. (2010). Checking consistency in mixed treatment comparison meta-analysis. Stat. Med..

[35] Phuapradit P., Varavithya W., Vathanophas K., Sangchai R., Podhipak A., Suthutvoravut U., Nopchinda S., Chantraruksa V., Haschke F. (1999). Reduction of Rotavirus Infection in Children Receiving Bifidobacteria-Supplemented Formula. J. Med. Assoc. Thai..

[36] Nopchinda S., Varavithya W., Phuapradit P., Sangchai R., Suthutvoravut U., Chantraruksa V., Haschke F. (2002). Effect of bifidobacterium Bb12 with or without Streptococcus thermophilus supplemented formula on nutritional status. J. Med. Assoc. Thai..

[37] Saavedra J.M., Abi-Hanna A., Moore N., Yolken R.H. (2004). Long-term consumption of infant formulas containing live probiotic bacteria: Tolerance and safety. Am. J. Clin. Nutr..

[38] Weizman Z., Asli G., Alsheikh A. (2005). Effect of a Probiotic Infant Formula on Infections in Child Care Centers. Pediatrics.

[39] Bakker-Zierikzee A.M., Alles M.S., Knol J., Kok F.J., Tolboom J.J., Bindels J.G. (2005). Effects of infant formula containing a mixture of galacto- and fructooligosaccharides or viable Bifidobacterium animalis on the intestinalmicroflora during the first 4 months of life. Br. J. Nutr..

[40] Weizman Z., Alsheikh A. (2006). Safety and Tolerance of a Probiotic Formula in Early Infancy Comparing Two Probiotic Agents: A Pilot Study. J. Am. Coll. Nutr..

[41] Brunser O., Figueroa G., Gotteland M., Haschke-Becher E., Magliola C., Rochat F., Cruchet S., Palframan R., Gibson G., Chauffard F. (2006). Effects of probiotic or prebiotic supplemented milk formulas on fecal microbiota composition of infants. Asia Pac. J. Clin. Nutr..

[42] Vendt N., Grünberg H., Tuure T., Malminiemi O., Wuolijoki E., Tillmann V., Sepp E., Korpela R. (2016). Growth during the first 6 months of life in infants using formula enriched with Lactobacillus rhamnosus GG: Double-blind, randomized trial. J. Hum. Nutr. Dietet..

[43] Mah K.W., Chin V.I., Wong W.S., Lay C., Tannock G.W., Shek L.P., Aw M.M., Chua K.Y., Wong H.B., Panchalingham A. (2007). Effect of a Milk Formula Containing Probiotics on the Fecal Microbiota of Asian Infants at Risk of Atopic Diseases. Pediatr. Res..

[44] Chouraqui J.P., Grathwohl D., Labaune J.M., Hascoet J.M., de Montgolfier I., Leclaire M., Giarre M., Steenhout P. (2008). Assessment of the safety, tolerance, and protective effect against diarrhea of infant formulas containing mixtures of probiotics or probiotics and prebiotics in a randomized controlled trial. Am. J. Clin. Nutr..

[45] Haschke-Becher E., Brunser O., Cruchet S., Gotteland M., Haschke F., Bachmann C. (2008). Urinary D-Lactate Excretion in Infants Receiving Lactobacillus johnsonii with Formula. Ann. Nutr. Metab..

[46] Gibson R.A., Barclay D., Marshall H., Moulin J., Maire J.C., Makrides M. (2009). Safety of supplementing infant formula with long-chain polyunsaturated fatty acids and Bifidobacterium lactis in term infants: A randomised controlled trial. Br. J. Nutr..

[47] Maldonado J., Lara-Villoslada F., Sierra S., Sempere L., Gómez M., Rodriguez J.M., Boza J., Xaus J., Olivares M. (2010). Safety and tolerance of the human milk probiotic strain Lactobacillus salivarius CECT5713 in 6-month-old children. Nutrition.

[48] Hascoët J.M., Hubert C., Rochat F., Legagneur H., Gaga S., Emady-Azar S., Steenhout P.G. (2011). Effect of Formula Composition on the Development of Infant Gut Microbiota. JPGN.

[49] López-Velázquez G., Díaz-García L., Anzo A., Parra-Ortiz M., Llamosas-Gallardo B., Ortiz-Hernández A.A., Mancilla-Ramírez J., Cruz-Rubio J.M., Gutiérrez-Castrellón P. (2013). Safety of a dual potential prebiotic system from Mexican agave incorporated to an inf ant formula for term newbom babies: A randomized controlled trial. Rev. Inv. Clin..

[50] Papagaroufalis K., Fotiou A., Egli D., Tran L.A., Steenhout P. (2014). A Randomized Double-Blind Controlled Safety Trial Evaluating d Lactic Acid Production in Healthy Infants Fed a Lactobacillus reuteri-containing Formula. Nutr. Metab. Insights.

[51] López-Velázquez G., Parra-Ortiz M., Mora Ide L., García-Torres I., Enríquez-Flores S., Alcántara-Ortigoza M.A., Angel A.G., Velázquez-Aragón J., Ortiz-Hernández R., Cruz-Rubio J.M. (2015). Effects of Fructans from Mexican Agave in Newborns Fed with Infant Formula: A Randomized Controlled Trial. Nutrients.

[52] Cooper P., Bolton K.D., Velaphi S., De Groot N., Emady-Azar S., Pecquet S., Steenhout P. (2016). Early Benefits of a Starter Formula Enriched in Prebiotics and Probiotics on the Gut Microbiota of Healthy Infants Born to HIV+ Mothers: A Randomized Double-Blind Controlled Trial. Clin. Med. Insights Pediatr..

[53] Kosuwon P., Lao-Araya M., Uthaisangsook S., Lay C., Bindels J., Knol J., Chatchatee P. (2018). A synbiotic mixture of scGOS/lcFOS and Bifidobacterium breve M-16V increases faecal Bifidobacterium in healthy young children. Benef. Microbes..

