# Supplementary material for: Health Effects of Infant Formula Supplemented with Probiotics or Synbiotics in Infants and Toddlers: Systematic Review with Network Meta-Analysis
Source: Nutrients. 2022 Dec 5;14(23):5175. doi: 10.3390/nu14235175 (PMC9739048; doi:10.3390/nu14235175)

Supplemental Information

1) **Supplemental Table S1**
2) **Supplemental Table S2**
3) **Supplemental Table S3**
4) **Supplemental Figure S1**
5) **Supplemental Figure S2**
6) **Supplemental Figure S3**
7) **Supplemental Figure S4**
8) **Supplemental Figure S5**
9) **Supplemental Figure S6**

Supplemental Table S1. Type of Probiotics included on the analysis

**Bifidobacteria**
B. lactis Bb12 (10^8 cfu/g)
B. lactis Bb12 1×10^7 cfu/
B. lactis Bb12 (3×10^7 cfu/g)
B. lactis Bb12 (6×10^9 cfu/100 ml) + GOS/FOS (6 g/l; 90%/10%)
B. lactis Bb12 3.85× 10^8 cfu + DHA and AA
B. lactis Bb12 + *S. thermophilus (*3×10^7 cfu/g)
B. lactis Bb12 + *S. thermophilus (*1×10^7 cfu/g)
B. lactis Bb12 + *S. thermophilus* 1×10^6 cfu/g
B. lactis Bb12 + *S. thermophilus* (dose not reported)
B. lactis strain CNCM-I-3446, 1 × 10^7 cfu/g + BMOS (GOS + 3′- and 6′-sialyllactose, 8 g/L)
B. breve CECT7263 (10^7 cfu/g)
B. breve M16-V (1.8x10^7 CFU/g) + GOS/FOS (9.5 g/l; 90%/10%)
B. longum BL999 2×10^7 cfu/g
B. longum BB536 1×10^7 cfu/g) + *Lactobacillus* GG 2×10^7 cfu
B. longum BL999 (1.29×10^8 cfu/100 ml) + LPR (6.45×10^8 cfu/100 ml)
B. longum BL999 (1.29×10^8 cfu/100 ml) + LPR (6.45×10^8 cfu/100 ml) + GOS/FOS (0.4 g/100 ml; 90%/10%)
B. longum BL999 (2.58×10^8 cfu/100 ml) + ST11 (2.58×10^8 cfu/100 ml + GOS/FOS (0.4 g/100 ml; 90%/10%)
B. longum BB536 1×107 cfu/g
B. infantis IM1 (10^7 cfu/g)
B. infantis R0033 (1.425×10^8 cfu) and *B. bifidum* R0071 (1.425×10^8 cfu), with 9.6×10^9 cfu of *L. helveticus* R0052
Bifidobacterium bifidum BF3, Bifidobacterium breve BR3, Bifidobacterium longum subspecies infantis BT1, and B. longum BG7. (1 x 107 CFU/g)

**Lactobacillus**
L. reuteri DSM 17938 1×10^7 cfu/g
L. reuteri DSM 17938 1.2×10^6cfu/ml
L. reuteri 1.2×10^9 cfu/l
Lactobacillus GG 10^7 cfu/g
L. johnsonii La1 10^8 cfu/g + FOS
L. salivarius CECT5713 2x10^6 cfu/g
L. rhamnosus GG 1x10^7 CFU/g + Inulin and fructan from agave (0.5g/100ml)
L. fermentum CECT5716 Lc40 (10^7 cfu/g)

Supplemental Table S2. Risk of bias evaluation for included RCTs

Reference	Adequate sequence generation?	Allocation concealment?	Blinding?	Incomplete outcome data addressed?	Free of selective reporting?	Free of other bias?
1999, Phaupradit[28]	unclear	unclear	unclear	unclear	unclear	unclear
2002, Nopchinda[29]	unclear	unclear	yes	no	unclear	unclear
2004, Saavedra[30]	yes	unclear	yes	yes	unclear	unclear
2005, Weizman[31]	yes	unclear	yes	yes	unclear	unclear
2005, Bakker-Zierikzee[32]	unclear	unclear	yes	no	unclear	unclear
2006, Weizman[33]	yes	unclear	yes	yes	unclear	unclear
2006, Brunser[34]	yes	yes	yes	yes	unclear	unclear
2006, Vendt[35]	yes	unclear	yes	yes	unclear	unclear
2007, Mah[36]	unclear	unclear	yes	unclear	unclear	unclear
2008, Chouraqui[37]	unclear	unclear	yes	yes	unclear	unclear
2008, Haschke-Becher[38]	unclear	unclear	yes	unclear	unclear	unclear
2009, Gibson[39]	yes	yes	yes	yes	unclear	unclear
2010, Maldonado[40]	unclear	unclear	yes	yes	unclear	unclear
2011, Hascoet[41]	unclear	unclear	unclear	no	unclear	unclear
2013 Gutiérrez-Castrellón[42]	yes	yes	yes	yes	yes	unclear
2014, Papagaroufalis[43]	yes	yes	yes	no	yes	unclear
2015 Gutiérrez-Castrellón[44]	yes	yes	yes	yes	yes	unclear
2016, Garcia Rodenas[6]	yes	yes	yes	yes	unclear	unclear
2016, Wu[7]	yes	yes	yes	no	unclear	unclear
2016, Cooper[45]	yes	yes	yes	yes	unclear	unclear
2016, Baglatzi[3]	yes	yes	yes	yes	yes	unclear
2017, Radke[12]	yes	yes	yes	yes	unclear	unclear
2018, Escribano[15]	yes	yes	yes	yes	yes	unclear
2018, Kosuwon[46]	yes	yes	yes	yes	unclear	unclear
2019, Xiao[16]	yes	yes	yes	yes	unclear	unclear
2019, Maldonado[17]	yes	yes	yes	yes	unclear	unclear

Supplemental Table S3. Excluded randomized controlled trials

**Reference**	**Reasons for exclusion**
Langhendries, 1995	Intervention: fermented formula
Kirjavainen, 2003	Population: infants with atopic dermatitis
Ziegler, 2003	Intervention: partially hydrolysed formula
Chouraqui, 2004	Intervention: fermented formula
Correa, 2005	Population: inpatients children receiving antibiotics (6-26 mo)
Brouwer, 2006	Population: infants with atopic dermatitis<br>Intervention: extensively hydrolysed whey formula with LGG
Rautava, 2006& 2009	Probiotics were not introduced during the manufacturing process, but thereafter, in capsules, the contents of which were supplemented to infant formula
Puccio, 2007	Intervention: formula with synbiotic
Hol, 2008	Population: infants with cow's milk allergy<br>Intervention: extensively hydrolysed formula with a combination of 2 probiotics (*Lactobacillus casei* CRL431 and *Bifidobacterium lactis* Bb-12)
Urban, 2008	Intervention: fermented formula
Velaphi, 2008	Intervention: fermented formula
West, 2008	Intervention: probiotic-supplemented cereal
Vlieger, 2009	Intervention: synbiotic formula vs prebiotic formula
Soh, 2009	Population: Children at risk (Family history) of allergic disorders
Scalabrin, 2009	Intervention: casein hydrolysate with LGG
Baldassare, 2010	Population: Infants with haematochezia (presumptive diagnosis of cow's milk allergic colitis)<br>Intervention: extensively hydrolysed casein formula with LGG
Cox, 2010	daily supplement of *Lactobacillus casei* subsp. *rhamnosus* (LGG)
Maldonado, 2010, 2012, 2015	Intervention: synbiotic formula vs formula with prebiotic
Dupont, 2010	Intervention: synbiotic formula vs normal formula
Gil-Campos, 2011	Intervention: synbiotic formula vs formula with prebiotic
Holscher, 2012	Intervention: partially hydrolysed whey formula
Muraro, 2012	Infants with cow's milk allergy
Marzotto, 2012	Intervention: yoghurt and fermented milk
Vandenplas, 2013	Infants with cow's milk allergy
Sarvetnick, 2013	Abstract from conference, outcome: changes in immune cell populations and activation markers
Berni-Canani, 2013	Non-randomised study
Bocquet, 2013	Intervention: formula with symbiotic versus formula with probiotic
Cohen, 2013	Intervention: synbiotic formula vs normal formula
Oswari, 2013	Intervention: formula with synbiotic
Cecola, 2015	Intervention: formula with probiotic versus formula with prebiotic
Baglatzi, 2015	Intervention: formula with low dose of *Bifidobacterium lactis* CNCM I-3446 versus formula with regular dose of *B lactis*.
Lee, 2015	Intervention: formula with probiotic vs formula with synbiotic
Fatheree, 2016	Population: infants with colic<br>Intervention: casein hydrolysate with LGG
Bazanella, 2016	Abstract from conference, outcome: structure and function of faecal microbiota
Fields, 2016	Intervention: extensively hydrolysed whey formula with *Bifidobacterium lactis*
Canani, 2016	Population: Infants with cow's milk allergy
Lingfen Xu, 2016	Population: Preterm infants
Canani, 2016	Population: Infants with cow's milk allergy
Scalabrin, 2017	Intervention: extensively hydrolysed formula
Indrio, 2017	Population: infants with functional regurgitation<br>Intervention: Partially hydrolysate whey infant formula
Soo Park, 2017	Population: children with rotavirus infection

Bazanella, 2017	Intervention: Comparison high dose vs low dose of the same probiotic
Vandenplas, 2017	Design: Open label, single arm
Guest, 2018	Population: Infants with cow's milk allergy
Korpela, 2018	Population: Intervention to mother during pregnancy and children
Candy, 2018	Population: Infants with cow's milk allergy
Wopereis, 2019	Population: Infants with cow's milk allergy
Cheng chi, 2019	Population: Low birth weight infants
Xiaonan Li, 2019	Intervention: Comparison with milk fat globule membrane enriched infant formula
Xuewei Cui, 2019	Population: Preterm infants
Basturk, 2019	Population: Infants with cow's milk allergy

Supplemental Figure S1. Network Meta-Analysis for probiotics in infant formula and colic; comparison adjusted funnel plot of multiple treatments.

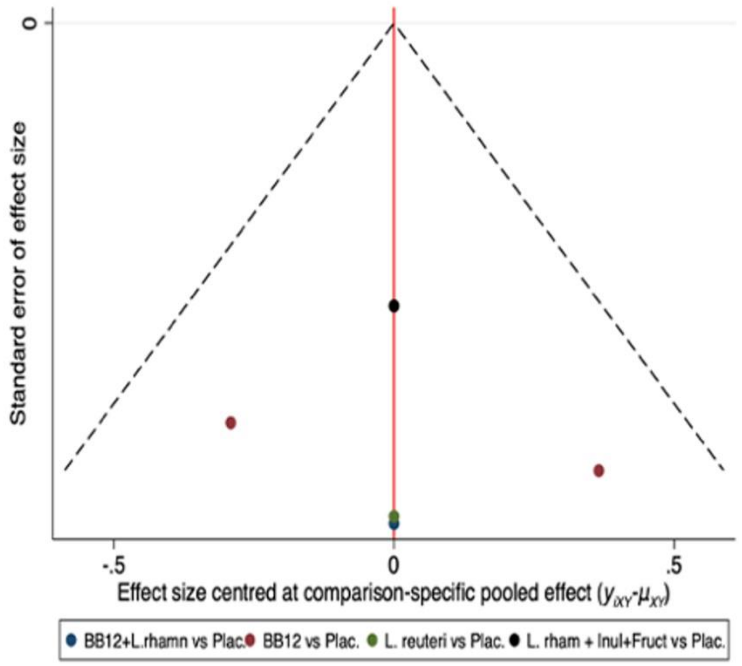

Supplemental Figure S2. Network Meta Analysis for probiotics in infant formula and diarrhoea; comparison adjusted funnel plot of multiple treatments.

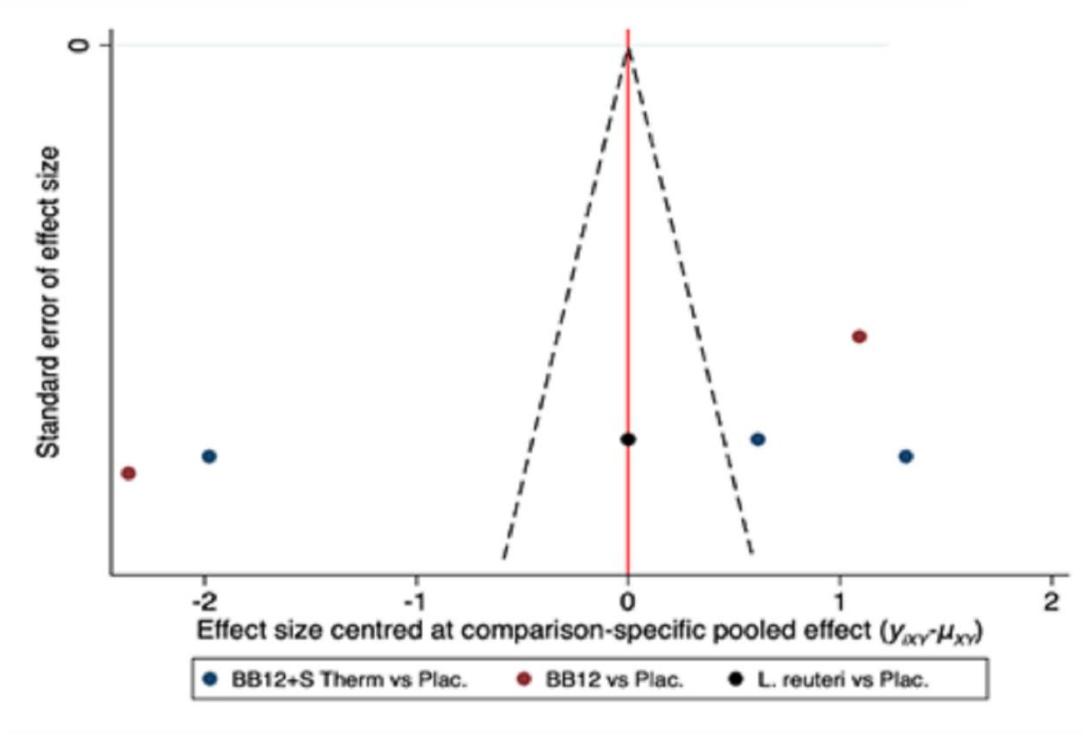

Supplemental Figure S3. Network Meta-Analysis for probiotics in infant formula and use of antibiotics; comparison adjusted funnel plot of multiple treatments.

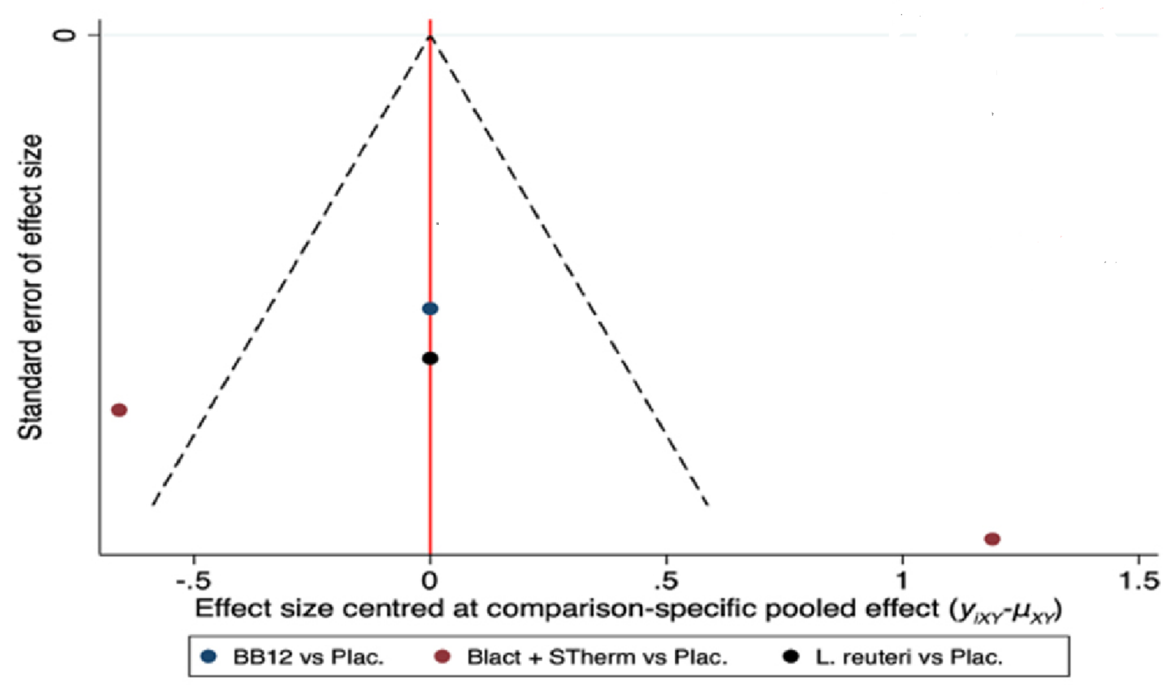

Supplemental Figure S4. Network Meta-Analysis for probiotics in infant formula and change on weight/height Z score; comparison adjusted funnel plot of multiple treatments.

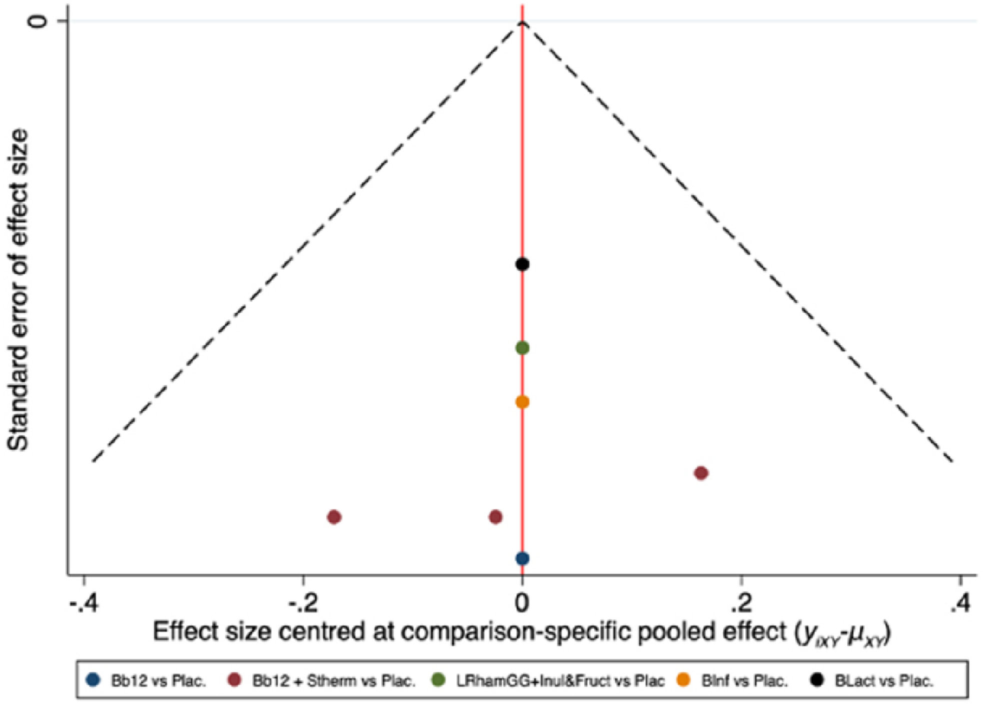

Supplemental Figure S5. Network meta-analysis for probiotics and change on fecal Bifidobacteria; comparison adjusted funnel plot of multiple treatments.

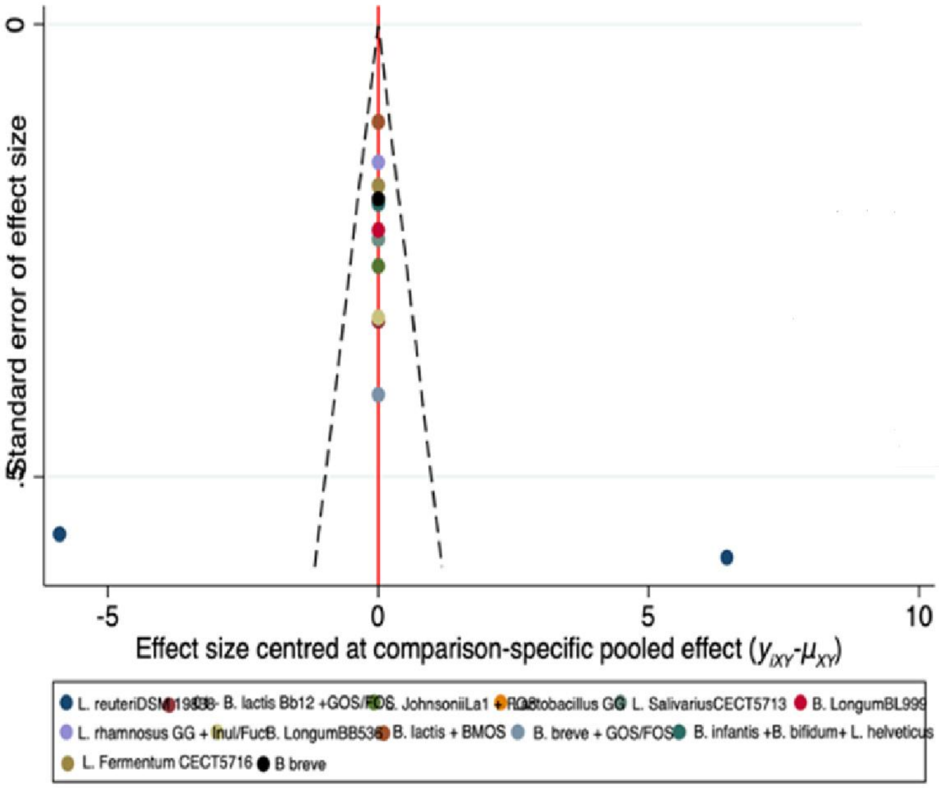

Supplemental Figure S6. Network meta-analysis for probiotics and change on fecal *Lactobacilli*; comparison adjusted funnel plot of multiple treatments.

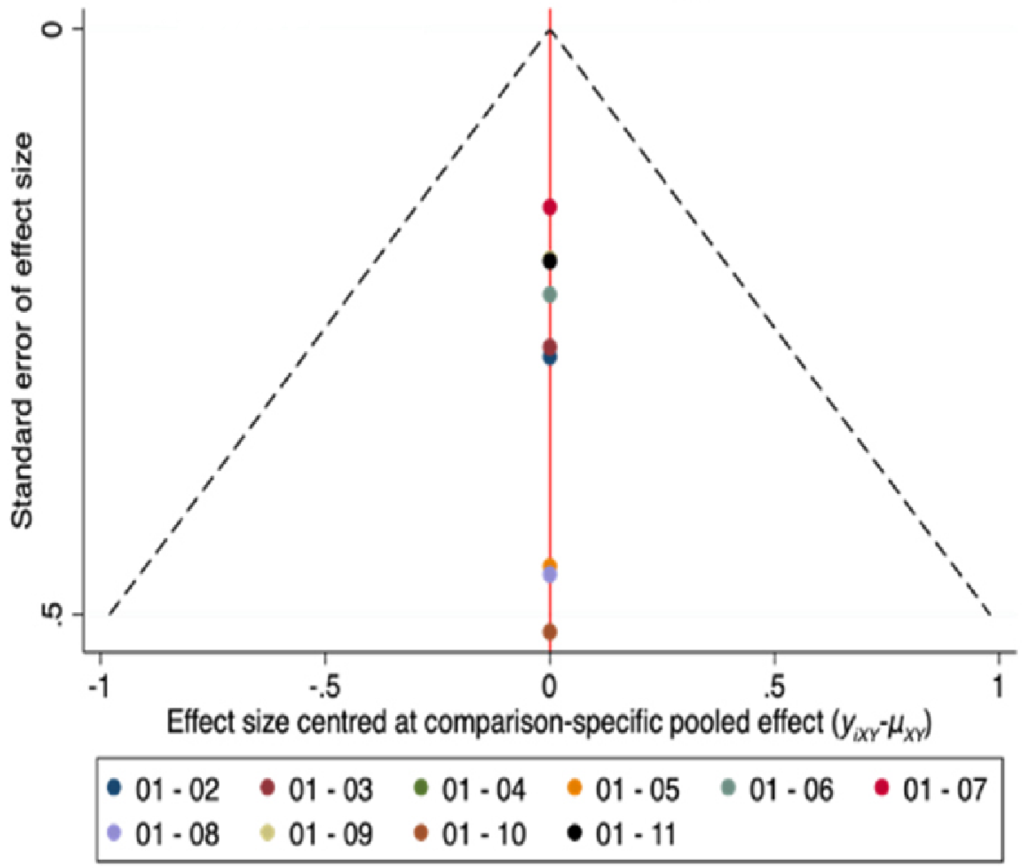

Supplement: Supplementary file 1 [file nutrients-14-05175-s001.zip › nutrients-2057725-supplementary.pdf]
